# Differential virulence between Asian and African lineages of Zika virus

**DOI:** 10.1371/journal.pntd.0005821

**Published:** 2017-09-21

**Authors:** Yannick Simonin, Debby van Riel, Philippe Van de Perre, Barry Rockx, Sara Salinas

**Affiliations:** 1 Pathogenesis and Control of Chronic Infections, INSERM/Université de Montpellier/ Etablissement Français du Sang, Inserm, Montpellier, France; 2 Department of Viroscience, Erasmus MC, Rotterdam, the Netherlands; 3 CHU de Montpellier, Department of Bacteriology-Virology, Montpellier, France; Baylor College of Medicine, UNITED STATES

Zika virus (ZIKV) is a small enveloped positive-sense single-stranded RNA virus belonging to the genus *Flavivirus* of the *Flaviviridae* family that has reemerged in recent years as a human pathogen with epidemic potential. To date, the ongoing epidemic in the Americas has led to tens of thousands of confirmed cases in Brazil alone (Pan American Health Organization Zika-Epidemiological Report, 2 March 2017). More concerning are the reports of severe neurological disorders such as Guillain-Barré syndrome and congenital Zika syndrome (microcephaly and other neurodevelopmental defects), which led the World Health Organization to declare the ZIKV epidemic as a Public Health Emergency of International Concern in 2016.

Phylogenetic analyses show that ZIKV can be classified into 2 main lineages: the African lineage and the Asian lineage, the latter of which is responsible for the recent epidemics. Because recent ZIKV infections were associated with the development of congenital and neurological disorders, a key question was raised as to whether Asian-lineage ZIKV strains were phenotypically different from the African lineage strains. It is well described that mutations acquired during *Flavivirus* evolution can alter their virulence and/or tropism [1]. ZIKV is primarily transmitted by the mosquito species *Aedes aegypti* and *Aedes albopictus*. Several studies show that vector transmission can differ between ZIKV strains, as the overall ZIKV infection prevalence and transmission rates of African strains may be higher in *A*. *aegypti* than Asian strains [2], suggesting that viral adaptation may have occurred—similar to a single mutation in the chikungunya virus envelope protein that affected vector specificity and epidemic potential.

During the recent outbreak, it became clear that ZIKV is sometimes able to cause a prolonged infection. Numerous studies showed that Asian-lineage strains isolated in South America replicate at low levels in tissues months after the initial infection. For example, viral genome can be found in stillborn babies who were infected early during gestation [3], weeks after initial infection in sperm, and in rhesus monkeys, ZIKV can be found in the cerebral spinal fluid (CSF) 42 days after infection, weeks after the virus was cleared from the blood [4]. However, to date, no data are available on the ability of African-lineage strains to cause prolonged infections, but early studies in 2016 suggested that an African ZIKV strain was highly pathogenic and led to cell death; when the first reports on a potential link between microcephaly and ZIKV emerged, laboratories started to study the effect of ZIKV on human neural precursor cells (hNPCs)/human neural stem cells (hNSCs) with MR766, the most available strain at the time (Fig 1 and Table 1). These first studies showed strong tropism and deleterious effects on NSC homeostasis and growth (e.g., [5]). However, some inconsistencies between this rather strong virulence and the long-term developmental effects associated with ZIKV infections led researchers to question the biological validity of this strain, which has been passaged many times in animals and in cells and acquired mutations and deletions at multiple positions during passaging (Table 1) [6–8]. The relevance of this strain was also questioned in a study that showed reduced toxicity and long-term persistence in human neural progenitors of an Asian-lineage ZIKV strain from Puerto Rico, which seemed more consistent with clinical manifestations [9]. Consequently, we compared the neurovirulence *ex vivo* of 2 low-passaged African- and Asian-lineage ZIKV strains [6]. The African ArB41644 strain was isolated in Central African Republic in 1989, whereas the Asian H/PF/2013 strain was isolated in 2013 during the French Polynesian epidemic (Table 1). We showed that the African ZIKV strain displayed a higher infectivity and replication rate in hNSCs than the Asian ZIKV strain. ArB41644 triggered more defects in proliferation and more apoptosis, which correlated with a stronger induction of the antiviral response [6]. In a subsequent study, we compared 2 Asian-lineage ZIKV strains (H/PF/2013 and ZIKVNL00013, isolated during the current epidemic in 2015) with 2 African-lineage ZIKV strains (MR766 and Uganda 927) in human neural progenitor cells and confirmed the phenotypic differences between ZIKV strains from the Asian and African lineages [10]. Both African strains infected more cells, replicated to higher titers, and induced early cell death more frequently than the Asian-lineage ZIKV strains [10]. The milder virulence of the Asian strains in these studies could be consistent with a persistent infection and effect of the Asian ZIKV lineage in developing neural cells. In addition, in dendritic cells, infection by African (MR766 and Dakar-1984) and Asian (PR-2015) strains led also to a difference in pathogenicity, as African strains triggered more cellular death [8]. Notably, this study also showed that despite differences in viral replication rates between the extensively passaged MR766 strain and the more recently isolated Dakar-1984 strain, the inductions of cell death were similar, suggesting that the ability to cause cell death seen in various cellular models with MR766 could still in certain circumstances be representative for African ZIKV lineage strains [8].

**Fig 1:**
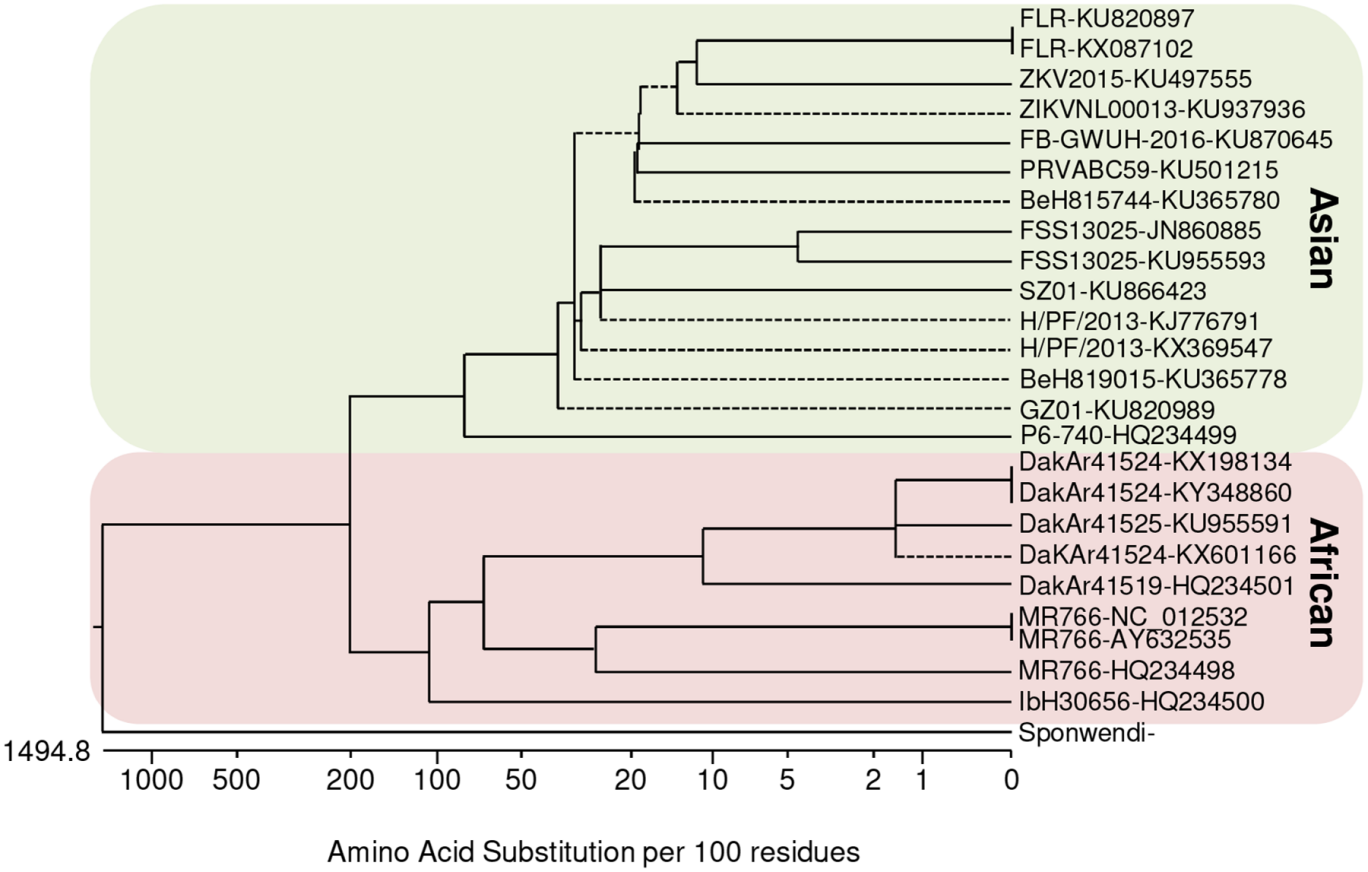
Phylogenetic analyses of ZIKV strains discussed.

**Table 1:** Passage history of ZIKV strains.

Lineage	Strain name	GenBank	Country of origin	Year	Reference	Passage histories
**African**	MR766	NC_012532; HQ234498.1; AY632535	Uganda	1947	[8]	Unknown + 1x Vero
					[11]	Not indicated
					[12]	Not indicated
					[13]	Not indicated
					[10]	Not indicated
					[14]	Not indicated
					[15]	Not indicated
					[16]	Not indicated
					[17]	Not indicated
					[18]	146x SM, 1x C6/36, 1x Vero
					[19]	146x SM, 3x Vero
					[20]	Not indicated
					[21]	Not indicated
	Uganda 976		Uganda	1962	[10]	6x Vero
	P6-740	HQ234499.1	Malaysia	1966	[8]	Unknown + 1x Vero
					[18]	6x SM, 1x BHK, 1x C6/36, 3x Vero
	IbH30656	HQ234500	Nigeria	1968	[16]	Not indicated
	Dakar41519	HQ234501	Senegal	1984	[14]	Not indicated
					[18]	1x AP61, 1x C6/36, 3x Vero
	Dakar 41671		Senegal	1984	[14]	Not indicated
	Dakar 41677		Senegal	1984	[14]	Not indicated
	DakAr41524	KX198134; KY348860; KX601166	Senegal	1984	[8]	Unknown + 1x Vero
	DakAr41525	KU955591	Senegal	1985	[22]	5x Vero, 1x C6/36, 2x Vero
					[2]	1x AP61, 1x C6/36
	ArB41644		Central African Republic	1989	[10]	5x Vero
					[6]	5x Vero
	HD78788	KF383039	Senegal	1991	[13]	Not indicated
	MP1751		Uganda	1962	[23]	3x SM,4x unknown methods, 1x Vero
**Asian**	FSS13025	KU955593; JN860885.1	Cambodia	2010	[15]	Not indicated
					[22]	1x AP-1, 1x C6/36, 5x Vero
					[2]	1x Vero, 1x C6/36
					[17]	Not indicated
					[18]	5x Vero
					[21]	Not indicated
	H/PF/2013	KX369547; KJ776791	French Polynesia	2013	[12]	Not indicated
					[10]	4x Vero
					[14]	Not indicated
					[24]	6x C6/36
					[6]	5x Vero
					[20]	Limited passages in Vero
	BeH815744	KU365780	Brazil	2015	[24]	3x C6/36
	BeH819015	KU365778.1	Brazil	2015	[20]	Not indicated
	FB-GWUH-2016	KU870645	Guatemala	2015	[12]	Not indicated
	FLR	KU820897; KX087102	Colombia	2015	[16]	Not indicated
	Mex1-7		Mexico	2015	[22]	3x Vero
					[2]	4x Vero
	PRVABC59	KU501215; KU501215.1	Puerto Rico	2015	[8]	Unknown + 1x Vero
					[23]	4x Vero
					[15]	Not indicated
					[16]	Not indicated
					[18]	3x Vero
					[19]	
	ZIKV^BR^		Brazil	2015	[11]	Not indicated
	ZKV2015	KU497555	Brazil	2015	[4]	Not indicated
	ZIKVNL00013	KU937936	Surinam	2016	[10]	4x Vero
	GZ01	KU820898	Venezuela	2016	[15]	Not indicated
	SZ01	KU866423	Samoa	2016	[16]	Not indicated

**Abbreviations:** BHK, baby hamster kidney, SM suckling mice

Since infection with ZIKV strains from the Asian lineage is associated with microcephaly in humans, it is likely that ZIKV crosses the placental barrier early in gestation, when the brain is starting to develop. Some studies showed that cells of the placenta are susceptible to ZIKV infection, in line with the congenital infections and neurological symptoms associated. Because infections early in gestation seem to be more linked to microcephaly [25], it was hypothesized that the placenta vulnerability could differ depending on the gestational stage. While the placental syncytiotrophoblast, which is found in mature placenta, appears mostly resistant to ZIKV, a study that compared an African strain (MR766) to an Asian strain (FSS13025, Cambodia) showed that embryonic stem cell (ESC)-derived trophoblasts, which recapitulate primitive placenta cells during implantation, are highly susceptible to ZIKV infection [17]. Interestingly, infection with the African strain again resulted in higher viral replication and cell lysis. These data might indicate that infection with African-lineage ZIKV strains—if they enter the fetus—could result in an early termination of pregnancy, while infection with an Asian-lineage ZIKV strain would be less destructive and more chronic, thereby allowing the pregnancy to continue, leading to the development of congenital malformations [17].

While these *ex vivo* data suggested that African ZIKV lineage strains are more virulent, it was important to confirm the difference in (neuro)virulence of these 2 lineages *in vivo*. Interferon-α/β receptor (*Ifnar*)^-/-^ mice injected subcutaneously with African ZIKV strains (MR766 and 3 strains from Senegal) or with the Asian-lineage H/PF/2013 strain all presented with weight loss and succumbed to the infection after 6 (for Senegal strains) to 10 (H/PF/2013) days. Interestingly, infection with the African-lineage strain MR766 resulted in 20% survival at 2 weeks [14]. Another study compared the symptoms induced by African (Uganda and Senegal) and Asian (Cambodia, Malaysia, and Puerto Rico) ZIKV strains in signal transducer and activator of transcription (*Stat2*)^-/-^ and *Ifnar*^-/-^ mice infected subcutaneously [18]. Importantly, infections with African strains resulted in more weight loss, mortality, and severe prolonged neurological symptoms compared to Asian strains. Moreover, induction of type 1 and 2 interferon (IFN) were higher following infection with African strains associated with enhanced levels of inflammatory cytokines such as interleukin 6 (IL6) or tumor necrosis factor (TNF). These observations were confirmed in another study showing that an African strain (MP1751) is pathogenic in A129 mice, contrary to an Asian strain (PRVABC59) that did not cause signs of illness [26]. These studies confirm the *ex vivo* results in neural and nonneural cells regarding the higher virulence of African strains and could suggest that different ZIKV strains may also display phenotypic differences in human subjects (Fig 1) (Table 2). Many of the findings from immunocompromised mice have been recapitulated in nonhuman primates, such as rhesus and cynomolgus macaques. The development of these different animal models led to new knowledge concerning the pathogenesis of ZIKV. However, these systems have limitations, in particular, studying viral pathogenesis using animals lacking a key component of antiviral immunity, which makes it still difficult to derive definitive conclusions regarding ZIKV virulence in humans. Therefore, many unanswered questions remain, in particular, regarding the mechanisms of host restriction, immune evasion, and inflammatory response as well as the long-term neurodevelopmental implications of congenital infection in humans.

**Table 2:** Comparative analyses of ZIKV African and Asian lineages.

	Model	*Lineage*: Strain	Findings	Reference
***Ex vivo***				
	Endothelial cells	*African*: MR766, IbH30656; *Asian*: PRVABC59, FLR	Asian-lineage strains replicate faster and isolates induced significant cell death compared to African-lineage strains.	[16]
	Astrocytes	*African*: HD78788, ArB41644; *Asian*: H/PF/2013	African-lineage strains led to a higher infection rate and viral production infection. Asian-lineage strain led to the expression of these innate immune response genes early, while their induction by the African strain was delayed.	[6][13]
	Neuronal stem cells	*African*: DakAr41525, MR766, ArB41644, Uganda 976; *Asian*: H/FP/2013, Mex1-7, FSS13025, FB-GWUH-2016, ZIKVNL00013	African-lineage strains led to a higher infection rate and virus production as well as stronger cell death and antiviral response compared to Asian-lineage strains. Protein 53 (p53) plays an important role in apoptosis induced by Asian-lineage strains but not by African-lineage strains.	[22][12][6]. [21][10]
	Cerebral organoids	*African*: MR766; *Asian*: ZIKV^BR^	Asian-lineage strain led to a higher viral production as well as stronger cell death compared to the African strain.	[11]
	Embryonic stem cell–derived trophoblast	*African*: MR766; *Asian*: FSS13025	African-lineage strain led to a higher viral production as well as stronger cell death compared to Asian-lineage strains.	[17]
	Monocyte-derived dendritic cells	*African*: DakAr41524, P6-740, MR766; *Asian*: PRVABC59	African-lineage strains led to a higher infection rate and viral production as well as stronger cell death and antiviral response compared to Asian-lineage strains.	[8]
	Neuronal cell lines (SK-N-SH, U87 MG)	*African*: *MR766*, *Uganda 976*; *Asian*: *H/FP/2013*, *ZIKVNL00013*	African-lineage strains led to a higher infection rate and virus production.	[10]
**In Vivo**				
	Mosquito: *Aedes aegypti*	*African*: DAKAR41525, MR766; *Asian*: MEX1–7, PRVABC59, GZ01, FSS13025, H/PF/2013, BeH815744	Infection prevalence and transmission rate of African-lineage strains is higher compared to Asian-lineage strains. Infection prevalence and transmission rate of Asian-lineage strains from the Americas are higher compared to Asian-lineage strains from Asia.	[15][24][2][19]
	Mouse: *A129*	*African*: MR766, P6-740, DAKAR41519,41667,41671,MP1751; *Asian*: H/PF/2013, PRVABC59, FSS13025, BeH819015, PRVABC59	African lineage is pathogenic whereas Asian lineage does not cause sign of illness.	[23]
	*Ifnar1*^*-/-*^ *Irf3*^*-/-*^ *Irf5*^*-/-*^ *Irf7*^*-/-*^		African-lineage strains induced higher mortality and severe neurological symptoms in a short period as compared to Asian-lineage strains.	[14]
	*Stat2*^*-/-*^ *Ifnar1*^*-/-*^		Among Asian-lineage strains, the Cambodia strain presented more severe symptoms including front limb paralysis and lethality, followed by the Malaysia and Puerto Rico strains. The Brazilian strain is least severe. The Uganda strain showed orders of magnitude higher viral RNA level compared to all other strains including Senegal, especially in brain tissue.	[18]
	*Ifngr1*^*-/-*^		Higher levels of interferon type I and type II and inflammatory cytokine induction by African-lineage strains as compared to the Asian-lineage strains.	[20]
	Nonhuman primate	*Asian*: ZKV2015, PRVABC59	No differences observed	[4]

However, one major challenge in interpreting and comparing the data from these studies is the lack of harmonization of the virus strains used. In this review alone, in which we focused on studies that compared multiple ZIKV strains, at least 12 African and 13 Asian strains were found (Fig 1, Tables 1 and 2). While most studies include the African lineage MR766 strain, this strain cannot be considered as a standard, as it has been extensively passaged and at least 3 MR766 strains exist with genetic differences, including a 4–6 codon deletion in the envelope (E) protein [7]. For other strains, multiple sequences exist, or in some cases, no sequence data are available (Table 1). Therefore, when comparing ZIKV lineages, the strains should be sequenced and experiments more standardized to allow proper comparison between studies. Ideally, more recent African ZIKV strains should become available (as the most recent isolate so far is from 1991) (Table 1).

In conclusion, the data reviewed here indicate that there are intrinsic differences in the pathogenicity/virulence of African- and Asian-lineage ZIKV strains. Whether these differences are responsible for differences in clinical presentations should be confirmed, but one can speculate that the phenotype of Asian ZIKV strains (lower infection rate, less virus production, and poor induction of early cell death) might contribute, at least in part, to the ability to cause persistent infections within the CNS of fetuses, while African-lineage ZIKV strains could result in more acute infection. However, even though *ex vivo* and *in vivo* data point towards a stronger virulence for African strains, it is still premature to conclude that if an African epidemic declares itself (due to an African ZIKV strain), neurological symptoms with similar or worse gravity than those observed in South America will be found. If confirmed, recent reports of African ZIKV strain infection and potential microcephaly in Guinea Bissau may be proof of what could be expected in this continent [27]. Determining the virulence factors for ZIKV using reverse genetic systems that have now been developed for both African and Asian strains will be crucial to identify the molecular and cellular mechanisms behind differences in ZIKV pathogenicity and also those of other emerging arboviruses.

## References

[1] Neal JW. Flaviviruses are neurotropic, but how do they invade the CNS? J Infect. 2014;69: 203–15. doi: 10.1016/j.jinf.2014.05.010 24880028 10.1016/j.jinf.2014.05.010

[2] Roundy CM, Azar SR, Rossi SL, Huang JH, Leal G, Yun R, et al Variation in *Aedes aegypti* Mosquito Competence for Zika Virus Transmission. Emerg Infect Dis. 2017;23: 625–632. doi: 10.3201/eid2304.161484 28287375 10.3201/eid2304.161484 PMC5367433

[3] Sarno M, Sacramento GA, Khouri R, do Ros?rio MS, Costa F, Archanjo G, et al Zika Virus Infection and Stillbirths: A Case of Hydrops Fetalis, Hydranencephaly and Fetal Demise. PLoS Negl Trop Dis. 2016;10: e0004517 doi: 10.1371/journal.pntd.0004517 26914330 10.1371/journal.pntd.0004517 PMC4767410

[4] Aid M, Abbink P, Larocca RA, Boyd M, Nityanandam R, Nanayakkara O, et al Zika Virus Persistence in the Central Nervous System and Lymph Nodes of Rhesus Monkeys. Cell. 2017;169: 610–620.e14 doi: 10.1016/j.cell.2017.04.008 28457610 10.1016/j.cell.2017.04.008 PMC5426912

[5] Garcez PP, Correia Loiola E, Madeiro da Costa R, Higa LM, Trindade P, Delvecchio R, et al Zika virus impairs growth in human neurospheres and brain organoids. Science (80). 2016;352: 816–818. 10.1126/science.aaf6116 27064148

[6] Simonin Y, Loustalot F, Desmetz C, Foulongne V, Constant O, Fournier-Wirth C, et al Zika Virus Strains Potentially Display Different Infectious Profiles in Human Neural Cells. EBioMedicine. 2016;12: 161–169. doi: 10.1016/j.ebiom.2016.09.020 27688094 10.1016/j.ebiom.2016.09.020 PMC5078617

[7] Haddow AD, Schuh AJ, Yasuda CY, Kasper MR, Heang V, Huy R, et al Genetic characterization of Zika virus strains: geographic expansion of the Asian lineage. PLoS Negl Trop Dis. 2012;6: e1477 doi: 10.1371/journal.pntd.0001477 22389730 10.1371/journal.pntd.0001477 PMC3289602

[8] Bowen JR, Quicke KM, Maddur MS, Neal JTO, Mcdonald E, Fedorova NB, et al Zika Virus Antagonizes Type I Interferon Responses during Infection of Human Dendritic Cells. PLoS Pathog. 2017; 1–30. 10.1371/journal.ppat.1006164 PMC5289613 28152048

[9] Hanners NW, Eitson JL, Usui N, Richardson RB, Wexler EM, Konopka G, et al Western Zika Virus in Human Fetal Neural Progenitors Persists Long Term with Partial Cytopathic and Limited Immunogenic Effects. Cell Rep. 2016;15: 2315–2322. doi: 10.1016/j.celrep.2016.05.075 27268504 10.1016/j.celrep.2016.05.075 PMC5645151

[10] Anfasa F, Siegers J, van der Kroeg M, Mumtaz N, Raj S, de Vrij F, et al Phenotypic differences between Asian and African lineage Zika viruses in human neural progenitor cells. mSphere. 2017;2: e00292–17. doi: 10.1128/mSphere.00292-17 28815211 10.1128/mSphere.00292-17 PMC5555676

[11] Cugola FR, Fernandes IR, Russo FB, Freitas BC, Dias JLM, Guimarães KP, et al The Brazilian Zika virus strain causes birth defects in experimental models. Nature. 2016;534: 267–7. doi: 10.1038/nature18296 27279226 10.1038/nature18296 PMC4902174

[12] Gabriel E, Ramani A, Karow U, Gottardo M, Natarajan K, Gooi LM, et al Recent Zika Virus Isolates Induce Premature Differentiation of Neural Progenitors in Human Brain Organoids. Cell Stem Cell. 2017;20: 397–406.e5. 28132835 10.1016/j.stem.2016.12.005

[13] Hamel R, Ferraris P, Wichit S, Diop F, Talignani L, Pompon J, et al African and Asian Zika virus strains differentially induce early antiviral responses in primary human astrocytes. Infect Genet Evol. 2017;49: 134–137. doi: 10.1016/j.meegid.2017.01.015 28095299 10.1016/j.meegid.2017.01.015

[14] Lazear H, Govero J, Smith A, Platt D, Fernandez E, Miner J, et al A Mouse Model of Zika Virus Pathogenesis. Cell Host Microbe. 2016;19: 720–730. doi: 10.1016/j.chom.2016.03.010 27066744 10.1016/j.chom.2016.03.010 PMC4866885

[15] Liu Y, Liu J, Du S, Shan C, Nie K, Zhang R, et al Evolutionary enhancement of Zika virus infectivity in Aedes aegypti mosquitoes. Nature. 2017;545: 482–486. doi: 10.1038/nature22365 28514450 10.1038/nature22365 PMC5885636

[16] Liu S, Delalio LJ, Isakson BE, Wang TT. AXL-Mediated Productive Infection of Human Endothelial Cells by Zika Virus. Circ Res. 2016;119: 1183–1189. doi: 10.1161/CIRCRESAHA.116.309866 27650556 10.1161/CIRCRESAHA.116.309866 PMC5215127

[17] Sheridan MA, Yunusov D, Balaraman V, Alexenko AP, Yabe S, Verjovski-Almeida S, et al Vulnerability of primitive human placental trophoblast to Zika virus. Proc Natl Acad Sci. 2017; 201616097. 10.1073/pnas.1616097114 PMC5338554 28193876

[18] Tripathi S, Balasubramaniam VRMT, Brown JA, Mena I, Grant A, Bardina S V., et al A novel Zika virus mouse model reveals strain specific differences in virus pathogenesis and host inflammatory immune responses. Pierson TC, editor. PLoS Pathog. 2017;13: e1006258 doi: 10.1371/journal.ppat.1006258 28278235 10.1371/journal.ppat.1006258 PMC5373643

[19] Weger-Lucarelli J, Rückert C, Chotiwan N, Nguyen C, Garcia Luna SM, Fauver JR, et al Vector Competence of American Mosquitoes for Three Strains of Zika Virus. PLoS Negl Trop Dis. 2016;10: e0005101 doi: 10.1371/journal.pntd.0005101 27783679 10.1371/journal.pntd.0005101 PMC5081193

[20] Widman DG, Young E, Yount BL, Plante KS, Gallichotte EN, Carbaugh DL, et al A Reverse Genetics Platform That Spans the Zika Virus Family Tree. MBio. 2017;8: e02014–16. doi: 10.1128/mBio.02014-16 28270583 10.1128/mBio.02014-16 PMC5340872

[21] Zhang F, Hammack C, Ogden SC, Cheng Y, Lee EM, Wen Z, et al Molecular signatures associated with ZIKV exposure in human cortical neural progenitors. Nucleic Acids Res. 2016;44: 8610–8620. doi: 10.1093/nar/gkw765 27580721 10.1093/nar/gkw765 PMC5063002

[22] McGrath EL, Rossi SL, Gao J, Widen SG, Grant AC, Dunn TJ, et al Differential Responses of Human Fetal Brain Neural Stem Cells to Zika Virus Infection. Stem cell reports. 2017;8: 715–727. doi: 10.1016/j.stemcr.2017.01.008 28216147 10.1016/j.stemcr.2017.01.008 PMC5355569

[23] Dowall SD, Graham VA, Rayner E, Atkinson B, Hall G, Watson RJ, et al A Susceptible Mouse Model for Zika Virus Infection. PLoS Negl Trop Dis. 2016;10: e0004658 doi: 10.1371/journal.pntd.0004658 27149521 10.1371/journal.pntd.0004658 PMC4858159

[24] Pompon J, Morales-Vargas R, Manuel M, Huat Tan C, Vial T, Hao Tan J, et al A Zika virus from America is more efficiently transmitted than an Asian virus by Aedes aegypti mosquitoes from Asia. Sci Rep. 2017;7: 1215 doi: 10.1038/s41598-017-01282-6 28450714 10.1038/s41598-017-01282-6 PMC5430906

[25] Kleber de Oliveira W, Cortez-Escalante J, De Oliveira WTGH, do Carmo GMI, Henriques CMP, Coelho GE, et al Increase in Reported Prevalence of Microcephaly in Infants Born to Women Living in Areas with Confirmed Zika Virus Transmission During the First Trimester of Pregnancy—Brazil, 2015. MMWR Morb Mortal Wkly Rep. 2016;65: 242–247. doi: 10.15585/mmwr.mm6509e2 26963593 10.15585/mmwr.mm6509e2

[26] Dowall SD, Graham VA, Rayner E, Hunter L, Atkinson B, Pearson G, et al Lineage-dependent differences in the disease progression of Zika virus infection in type-I interferon receptor knockout (A129) mice. PLoS Negl Trop Dis. 2017;11: e0005704 doi: 10.1371/journal.pntd.0005704 28672028 10.1371/journal.pntd.0005704 PMC5510909

[27] Nutt C, Adams P. Zika in Africa—the invisible epidemic? Lancet. 2017;389: 1595–1596. doi: 10.1016/S0140-6736(17)31051-6 28593839 10.1016/S0140-6736(17)31051-6

